# Exploring subcellular responses of prostate cancer cells to X-ray exposure by Raman mapping

**DOI:** 10.1038/s41598-019-45179-y

**Published:** 2019-06-18

**Authors:** Maciej Roman, Tomasz P. Wrobel, Agnieszka Panek, Esen Efeoglu, Joanna Wiltowska-Zuber, Czeslawa Paluszkiewicz, Hugh J. Byrne, Wojciech M. Kwiatek

**Affiliations:** 1 Institute of Nuclear Physics Polish Academy of Sciences, PL-31342 Krakow, Poland; 2 FOCAS Research Institute, Technological University Dublin, Kevin Street, Dublin, 8 Ireland

**Keywords:** Radiotherapy, Biophysical chemistry

## Abstract

Understanding the response of cancer cells to ionising radiation is a crucial step in modern radiotherapy. Raman microspectroscopy, together with Partial Least Squares Regression (PLSR) analysis has been shown to be a powerful tool for monitoring biochemical changes of irradiated cells on the subcellular level. However, to date, the majority of Raman studies have been performed using a single spectrum per cell, giving a limited view of the total biochemical response of the cell. In the current study, Raman mapping of the whole cell area was undertaken to ensure a more comprehensive understanding of the changes induced by X-ray radiation. On the basis of the collected Raman spectral maps, PLSR models were constructed to elucidate the time-dependent evolution of chemical changes induced in cells by irradiation, and the performance of PLSR models based on whole cell averages as compared to those based on average Raman spectra of cytoplasm and nuclear region. On the other hand, prediction of X-ray doses for individual cellular components showed that cytoplasmic and nuclear regions should be analysed separately. Finally, the advantage of the mapping technique over single point measurements was verified by a comparison of the corresponding PLSR models.

## Introduction

Prostate cancer is the second leading cause of cancer death in men^1^. It grows locally, but also possesses the ability to metastasise to distant organs in the body such as brain or lungs. Although initial response rates to the available treatments are high, a large number of cancer patients suffers relapse years or even decades later^2,3^, thus representing a serious therapeutic challenge. Recently, many treatment opportunities for cancer have become widely available, *i.e*.: chemotherapy, surgery, palliative care, and radiation therapy (also called ‘radiotherapy’). Radiotherapy remains an important element of the cancer treatment as it offers survival and palliative benefits^4–6^. For many patients with inoperable tumours, radiation therapy is the only option. Furthermore, patients who are incompletely resected or have recurrent tumours after surgery are usually treated with radiation as an adjuvant therapy^7^. This kind of therapy destroys cancer by delivering radiation of high energy to the tumour volume, while sparing the normal tissue. The biological effectiveness of ionizing radiation depends on the radiation source (total dose, fractionation rate, linear energy transfer (LET)) and radiosensitivity of the target (cells or tissues)^8^. The main goal of radiation treatment is the induction of cell or tissue damage beyond repair threshold and consequently triggering death pathways. In this context, DNA damage is a critical target of radiation action. Radiation induces ionisation that may directly influence the structure of critical cellular components and lead to serious damage. Additionally, ionisation can act indirectly by generating free radicals^9^. Such highly reactive species are derived from the ionisation or excitation of water present in the cells (water content in a cell is *ca*. 80%)^9^. For ionising radiation such as low LET X-rays and gamma-rays, the indirect effects cause *ca*. 60% of total cellular damage^10^. One kind of radiation damage is the production of double strand breaks (DSBs), which is the most lethal type of DNA modification. If not repaired, it usually leads to cell death. On the other hand, response mechanisms of DNA damage play a key role in cell defence against radiation-induced damage serving two important functions: cell survival and the maintenance of genomic stability. As can be seen, the mechanism of cell response to irradiation can be complex. Thus, identifying the mechanisms of radiation induced cell responses, both in terms of damage and repair, has potential clinical implications for improving outcomes with radiation therapy.

Most studies of radiation effects have been centred on the detection of DNA damage, usually analysed by biochemical methods, such as: comet assay, electrophoresis, chromatography, γH2aX test, Fluorescent *in Situ* Hybridisation, and biochemical staining^11,12^. However, such methods can influence the composition of biological materials. Multiple preparation procedures and the use of reactive chemicals may lead to significant changes in the biomolecules structure. Furthermore, biochemical techniques are not usually designed for studies on radiation-induced effects at the single cell or subcellular level. Thus, application of complementary methods to investigate the cell response caused by ionising radiation at the molecular level is in high demand^12^.

Spectroscopic methods are highly suitable for the detection and characterisation of complex biological systems^13,14^. In particular, Raman spectroscopy has shown promise, as it provides detailed molecular information about the sample and can easily detect structural changes^15,16^. Furthermore, as an optical technique, this method is non-invasive and non-destructive, allowing the analysis of live cells or tissues without perturbation of the sample^17–19^. The use of high-power focusing optics in a confocal microscopic mode can provide spatial resolutions lower than 1 μm, which is well below the typical size of a human cell (10–50 μm diameter). Raman spectroscopy is label free, and thus has the advantage over other microscopic profiling techniques that it can simultaneously detect a variety of molecular structures (proteins, nucleic acids (DNA and RNA), and lipids) in a single acquisition. Thus, it is possible to profile the composition of subcellular structures across the whole cell using mapping techniques, and changes to them due to exogenous agents such as nanoparticles^20^, drugs^21,22^, or radiation^23^.

Raman spectroscopy has been widely applied for biochemical analysis of DNA^24–29^, cells^30–34^, and tissues^35–38^ treated by various types of radiation. Early Raman studies on radiation effects were based on DNA^24–29^, showing the usefulness of this method for investigation of radiation-induced damage in biomolecules. Further studies based on cells and tissues have confirmed the utility of Raman spectroscopy in radiation research. For instance, Matthews *et al*. have applied principal components analysis of Raman signatures for differentiation between unirradiated and irradiated prostate cancer cells (DU145 cell line)^30^. A single fraction of 6 MV photons at doses in the range of 15–50 Gy was seen to induce biochemical changes in the cells, including differences in lipids, nucleic acids, amino acids and conformational protein structures^30^. A further study by the same research group focused on radiation-based changes in cells that vary by tissue of origin (prostate, breast, lung)^31^. The authors concluded that cell response to the radiation is different across the studied cell lines and depends on cell radiosensitivity^31^. A similar approach has been applied by Harder *et al*.^34^. It has been shown that irradiation of selected lung and breast cell lines resulted in accumulation of glycogen, whereas irradiation of the prostate LNCaP cell line showed a membrane phospholipid-related radiation response^34^. As can be seen, literature reports indicate a wide diversity of studies on radiation-induced cell response. However, all cited studies have been performed by single acquisition per cell with no discrimination between individual cell components (nucleus, cytoplasm, cell organelles). Although this is well justified by the relatively slow speed of Raman data acquisition, there is an inherent risk of omitting important information when only a small and random subset of a cell area is probed, as opposed to imaging the whole cell. Furthermore, Raman mapping shows the potential as an *in vitro* assay, for screening drug efficacies and modes of action^21,22,39,40^. There are only a few works based on Raman imaging of radiation-based changes in biological systems^41,42^, but none of them have attempted to assess the subcellular response of the cells. In order to address this gap and investigate whether mapping of the whole cell provides more insight into chemical changes, in our study we applied Raman microspectroscopic mapping to monitor subcellular changes induced by ionising radiation (X-ray) in PC-3 prostate cancer cells. Since differences between average Raman spectra of the cells treated with an increasing X-ray dose are minor (see Supplementary Figs S1–S3), Partial Least Squares Regression (PLSR) analysis was employed to construct appropriate prediction models. The models relate the predicted dose to the actual dose employed, at different time points after irradiation, the regression coefficient, or PLSR β-vector, revealing the spectral signatures which can be associated with biochemical changes at the cellular and/or subcellular level. The different analytical approaches of (i) averaging the spectral responses over the whole cell (ii) probing the different responses at a subcellular level were then compared and contrasted.

## Results and Discussion

### Cell response to X-ray irradiation

X-ray treated prostate cancer (PC-3) cells were spectroscopically mapped to monitor the subcellular response to the ionising radiation. Cells for different doses of radiation were incubated with different times to observe initial chemical changes due to irradiation and to monitor the subsequent cellular response.

**Figure 1:**
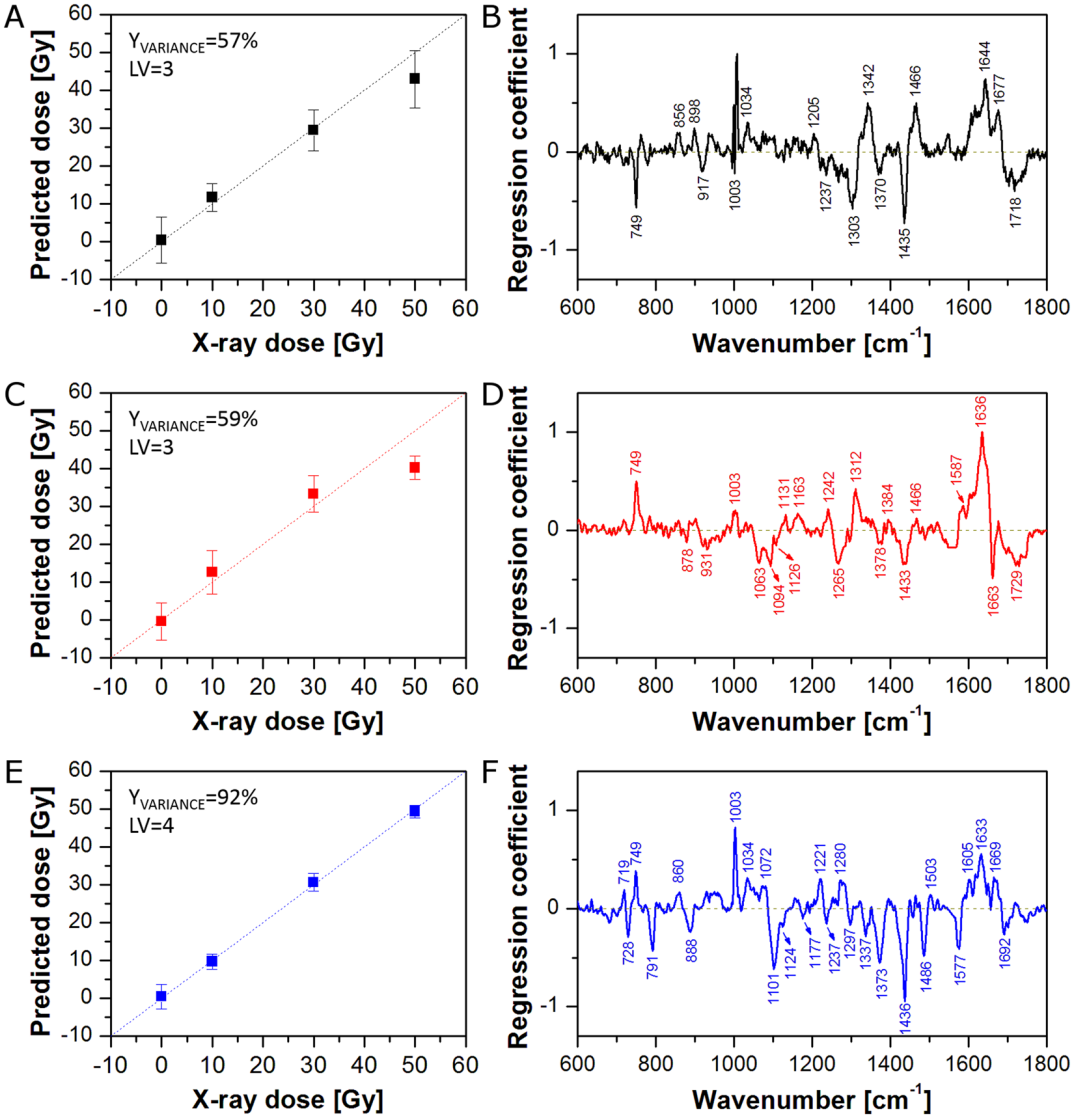
PLSR models for cells fixed 0 h (**A,B**), 24 h (**C,D**), and 48 h (**E,F**) after irradiation: predicted versus applied X-ray dose (**A,C,E**) and regression coefficient β plots (**B,D,F**). Average spectra of the whole cell were used as the PLSR model input.

In the first approach, Raman spectra from all pixels (nuclear region and cytoplasm) were averaged to obtain one mean spectrum of each cell. PLSR models for each of the incubation time dataset are presented in Fig. 1. As can be seen, all PLSR models (0 h, 24 h, and 48 h) show good linearity (X-ray dose prediction), although, the 0 h and 24 h models (Fig. 1A,C) deviate somewhat from linearity, and therefore predict lower doses for cells treated with 50 Gy.

Regression coefficient (β) plots (Fig. 1B,D,F) show spectral changes related to the increase of X-ray dose, calculated for the corresponding PLSR models. Positive features indicate an increase in the intensity of a specific vibrational response, as a result of changes in biomolecular content, morphology or conformation. Figure 1B shows the PLSR β plot for the 0 h model. As can be seen, most spectral changes are related to proteins (856, 1003, 1034, 1205, 1237, 1342, 1466, 1644, 1677 cm^−1^) and lipids (1303, 1435, 1718 cm^−1^)^43–47^. The feature at (749 cm^−1^) is most commonly associated with cytochrome C (cytoplasmic region only)^48,49^ although no strong features of conjugated C=C (*ca*. 1585 cm^−1^) are observable. On the other hand, the PLSR β plot for the 24 h model (Fig. 1D) shows bands characteristic for cytochrome C (749, 1587 cm^−1^), nucleic acids (1094, 1378 cm^−1^), proteins (931, 1003, 1131, 1163, 1242, 1312, 1466, 1636 cm^−1^), and lipids (878, 1063, 1127, 1265, 1433, 1663, 1729 cm^−1^)^43–47,50,51^. Finally, Fig. 1F presents the PLSR β plot for the 48 h model, which shows bands characteristic for choline (719 cm^−1^), cytochrome C (749 cm^−1^), nucleic acids (728, 791, 1101, 1337, 1373, 1486, 1577 cm^−1^), proteins (1003, 1034, 1072, 1221, 1237, 1605, 1633, 1669 cm^−1^), and lipids (888, 1124, 1297, 1436 cm^−1^)^43–51^. Spectral changes observed for the 0 h model come from direct damage and early cell response, whereas the spectral pattern observed in the PLSR β plots for the 24 h and 48 h models can be assigned to later cell responses to the radiation. Analysis of positive and negative bands leads to the conclusions that irradiation of cells results in significant changes in the content/conformation of proteins as well as nucleic acids and lipids. The results seem to be intuitive, as higher content of proteins can be explained by higher protein production as a consequence of execution of repair processes^52–55^, lower content of nucleic acids comes directly from DNA/RNA damage, and lower content of lipids seems to be a result of stress responses of cells. Stress induced release of fatty acids from the stored lipid droplets (LDs) in cells provides energy which subsequently promote cell survival^56^. Some indications of signatures of cytochrome C are apparent, in both negative and positive senses, and these will be explored further in the ‘Raman mapping *versus* single point measurements’ section.

**Figure 2:**
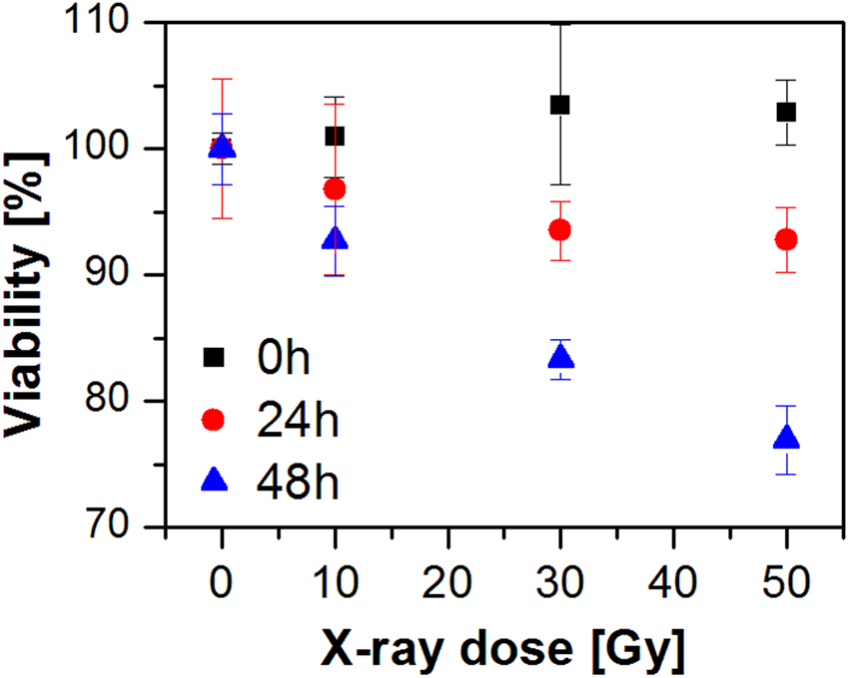
Results of a cell metabolic activity test (MTT assay) for 0 h, 24 h, and 48 h post-irradiation.

The results of the spectroscopic investigation have been compared to the cell metabolic activity test (MTT assay, Fig. 2). As can be seen in Fig. 2, cells fixed immediately after irradiation (0 h) show no reduction in metabolic activity. In this case, the cells have had no time to respond to the irradiation and no post-irradiation effect is observed. On the other hand, spectroscopic data show significant dependence on the X-ray dose in the 0 h model (Fig. 1A). It seems that Raman spectroscopy detects chemical changes caused directly by radiation and early cell response, which are not related to the later cell response observed subsequent to irradiation (regression coefficient plots for the 0 h and 24 h/48 h models show distinct spectral differences – compare Fig. 1B,D,F). In a similar fashion, Raman microspectroscopy has been demonstrated to differentiate the initial chemical binding effects of chemotherapeutic agents, from the subsequent cellular metabolic responses^57^. 24 h after irradiation, the viability decreases slightly with a dose, showing weak destructive radiation effects on cells. A similar situation can be observed for 48 h. The decrease of cell metabolic activity with X-ray dose is not significant but more prominent than for 24 h. Although the results of the MTT assay for 24 h and 48 h confirm cells respond to irradiation, this test is limited to the longer incubation times (24 h and 48 h) and does not provide any chemical information about the response. On the other hand, the results of Raman microspectroscopy show significant radiation-induced changes in the cell biochemistry even at the 0 h timepoint. Furthermore, the identified responses have differing subcellular origins, and the response rates of, for example, DNA damage in the nucleus and cytochrome C signatures potentially associated with mitochondrial damage in the cytoplasm could differ significantly. Therefore, it is of interest to probe the spectroscopic responses at a subcellular level.

### Subcellular response to X-ray irradiation

**Figure 3:**
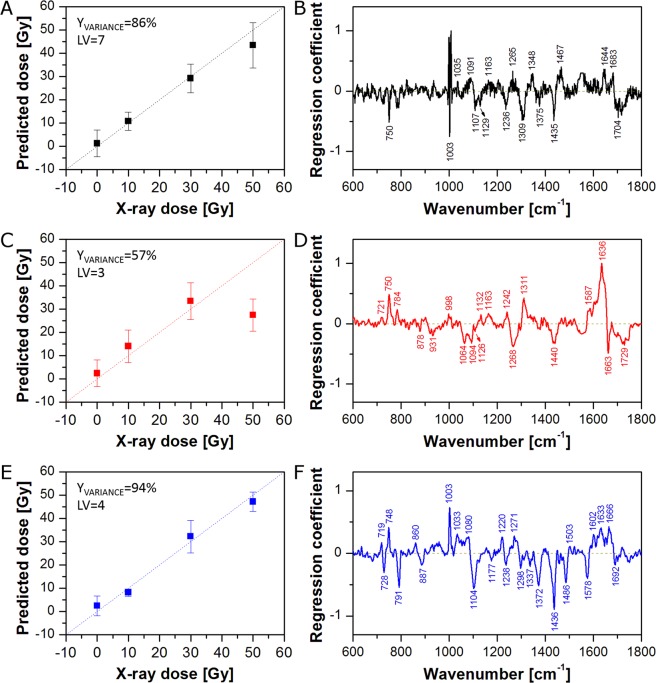
PLSR models for the cytoplasmic region of cells fixed 0 h (**A,B**), 24 h (**C,D**), and 48 h (**E,F**) after irradiation: predicted versus applied X-ray dose (**A,C,E**) and regression coefficient β plots (**B,D,F**). Average spectra of the cytoplasmic region were used as the PLSR model input.

**Figure 4:**
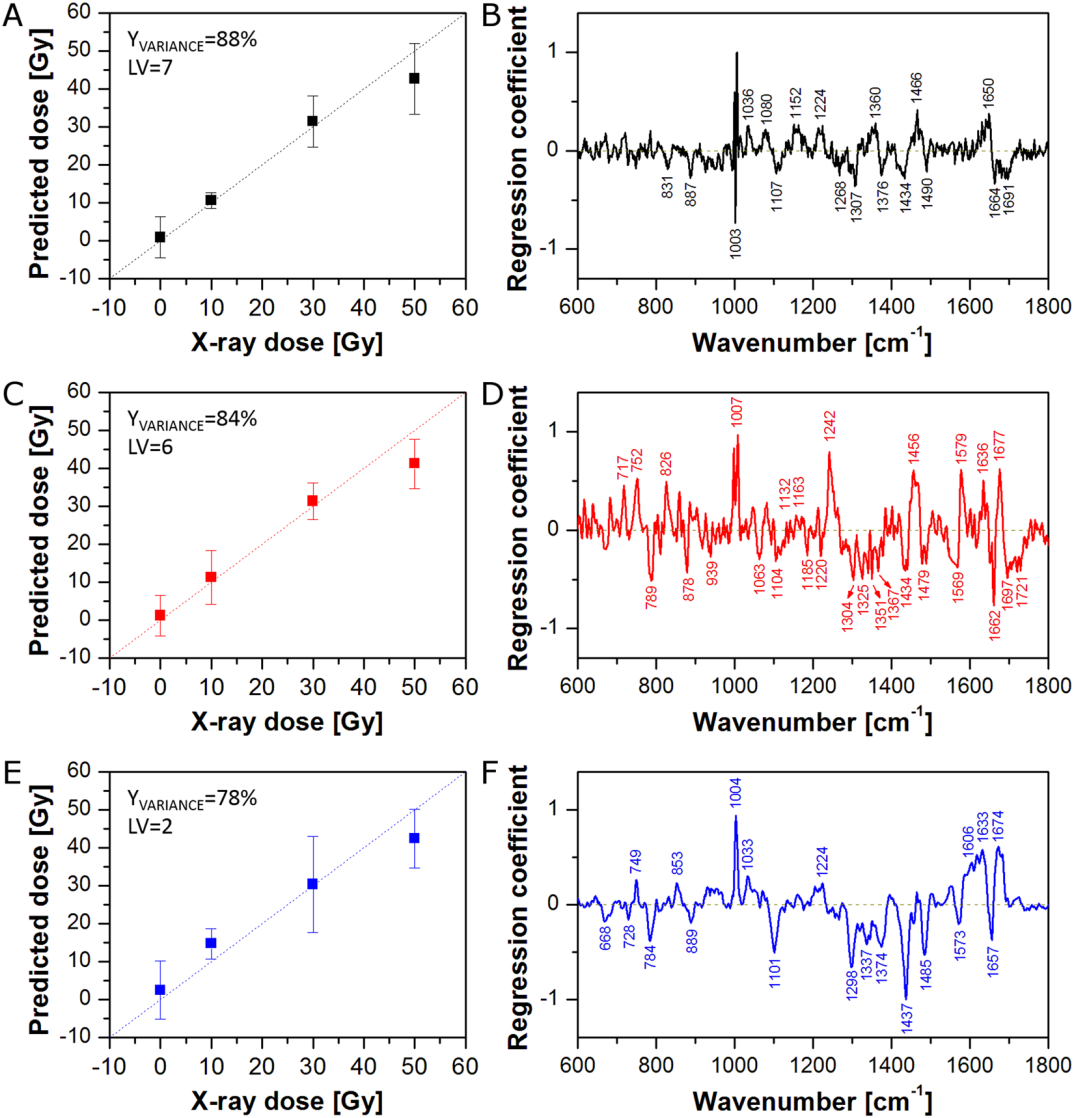
PLSR models for the nuclear region of cells fixed 0 h (**A,B**), 24 h (**C,D**), and 48 h (**E,F**) after irradiation: predicted versus applied X-ray dose (**A,C,E**) and regression coefficient β plots (**B,D,F**). Average spectra of the nuclear region were used as the PLSR model input.

It is known that the nucleus and cytoplasm are chemically distinct and may have different response to radiation. Therefore, in the second approach, Raman spectra from cytoplasm and the nuclear region were averaged separately to obtain one mean spectrum of cytoplasm and one mean spectrum of the nuclear region for each cell. PLSR models for cytoplasm and the nuclear region of cells fixed 0 h, 24 h, and 48 h after irradiation are presented in Fig. 3 and Fig. 4, respectively. As can be seen, all PLSR models show good X-ray dose prediction. As was observed for the PLSR model based on the whole cell (Fig. 1A), although the cells do not have time to respond to the radiation, PLSR models for cytoplasm (Fig. 3A) and the nuclear region (Fig. 4A) of cells fixed immediately after irradiation (0 h) exhibit clear correlation between applied and predicted X-ray dose. The PLSR β plot for cytoplasm (Fig. 3B) shows a very similar spectral pattern to that observed for the PLSR model for the whole cell (Fig. 1B). Furthermore, despite some differences in band intensities, the PLSR β plot for the nuclear region (Fig. 4B) exhibits a similar spectral pattern as well. This suggests that the reactions of cytoplasm and nucleus to the irradiation are similar. For the 24 h models, correlation between applied and predicted X-ray dose is quite good, although, the PLSR model for cytoplasm (Fig. 3C) shows a significant shift of 50 Gy samples towards lower X-ray doses. Nevertheless, the PLSR β plot for cytoplasm (Fig. 3D) exhibits an almost identical spectral pattern as observed for the whole cell (Fig. 1D). On the other hand, the PLSR β plot for the nuclear region (Fig. 4D) shows a completely different spectral pattern. It suggests a dominant influence of cytoplasm pixels on PLSR models for the whole cell. It is obvious that, due to the ratio of nucleus size to the whole cell area (in this cell line), the number of cytoplasm pixels exceeds those of the nuclear region (see Supplementary Fig. S4). The comparison highlights that chemical changes in the nucleus can easily go unnoticed in the PLSR models of the whole cell. Thus, it is vitally important to analyse cytoplasmic and nuclear regions independently. Finally, the best correlations between applied and predicted X-ray dose were achieved for cells fixed 48 h after irradiation (Figs 1E, 3E and 4E). Furthermore, the PLSR β plots for the whole cell (Fig. 1F), cytoplasm (Fig. 3F), and the nuclear region (Fig. 4F) show almost the same spectral pattern, at this post-irradiation timepoint. However, the PLSR β plot for the nuclear region (Fig. 4F) shows no choline band (719 cm^−1^), a difference in position of the DNA band (784 cm^−1^ for nucleus *versus* 791 cm^−1^ for cytoplasm), and an additional band at 1657 cm^−1^ (C=C stretching vibrations). Furthermore, the presence of a band at *ca.* 750 cm^−1^ in the PLSR β plots for 24 h and 48 h (Fig. 4D,F, respectively) can be confusing. In the first case (24 h, Fig. 4D), this band is slightly shifted towards higher wavenumbers (752 cm^−1^), commonly assigned to tryptophan^47^. However, the PLSR β plot for 48 h shows this band at the position of the cytochrome C band (749 cm^−1^), suggesting it could derive from mitochondria located above and below the nucleus which contribute to the average spectrum from the nuclear region. The difference in position of the DNA band seems particularly interesting. The DNA band at 784 cm^−1^ originates from ring breathing vibrations of DNA base pairs, whereas the band at 791 cm^−1^ comes from O–P–O stretching vibrations of the DNA backbone^43,44,50,51^. It suggests different damage in DNA molecules located in nucleus and cytoplasm region. Finally, high predictive power of PLSR models for 48 h suggests that changes observed in cells after 48 h reflect to a great extent the real chemical changes in PC-3 cancer cells caused by X-ray irradiation.

### Subcellular dose prediction

**Figure 5:**
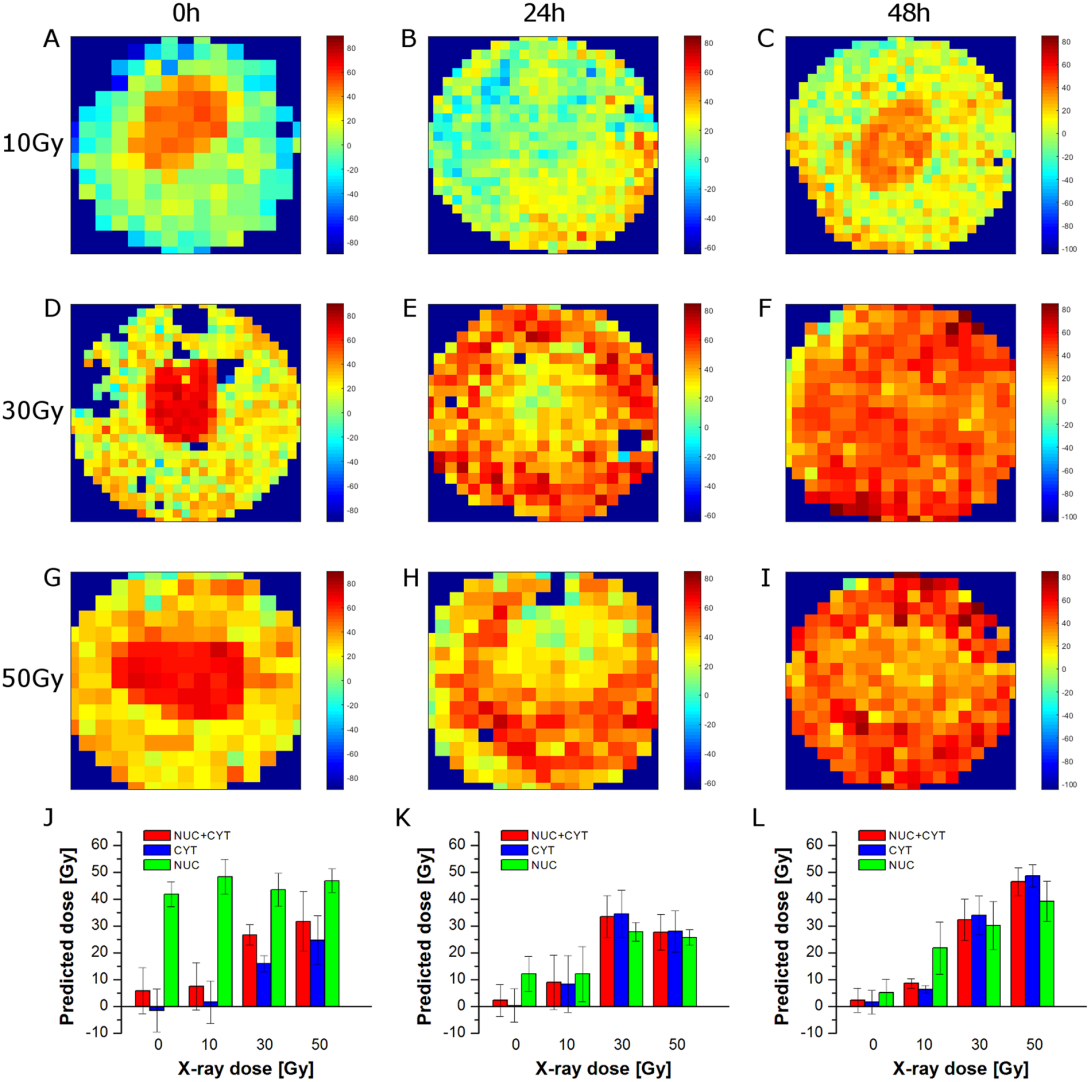
Selected Raman maps of predicted X-ray dose for 0 h (**A,D,G**), 24 h (**B,E,H**), and 48 h (**C,F,I**) calculated on the basis of PLSR models for the whole cell (from Fig. 1). Raman maps are presented for 10 Gy (**A–C**), 30 Gy (**D–F**), and 50 Gy (**G–I**). Bar graphs of predicted X-ray dose for 0 h (**J**), 24 h (**K**), and 48 h (**L**) calculated on the basis of the PLSR models for the average spectra of the whole cell (Nuc + Cyt), cytoplasm (Cyt), and the nuclear region (Nuc).

PLSR models based on average spectra of the whole cell (Fig. 1) or the cytoplasmic and nuclear regions (Figs 3 and 4) show spectral features responsible for radiation effects on PC-3 cancer cells. However, Raman mapping can provide spatial information about localised biochemical changes caused by external factors. Thus, on the basis of the calculated PLSR models, it is possible to predict X-ray dose delivered to all parts of the cells. Figure 5 shows the spatial distribution of the predicted X-ray dose based on the PLSR models (shown in Fig. 1) for the whole cell irradiated after 0 h (Fig. 5A,D,G), 24 h (Fig. 5B,E,H), and 48 h (Fig. 5C,F,I) with 10 Gy (Fig. 5A–C), 30 Gy (Fig. 5C,E,F), and 50 Gy (Fig. 5G,H,I). As can be seen, for 0 h, higher dose is predicted for the nuclear region than for the cytoplasm. This can be easily seen in Fig. 5J, in which the average predicted doses for the whole cell (Nuc + Cyt), cytoplasm (Cyt), and the nuclear region (Nuc) are gathered. Indeed, the average predicted dose for all applied X-ray doses is much higher for the nuclear region than for the cytoplasm. On the other hand, cells fixed 24 h after irradiation show much higher predicted dose in the nuclear region for 0 Gy and 10 Gy, whereas higher predicted dose in the cytoplasm for 30 Gy and 50 Gy (Fig. 5B,E,H,K). A similar pattern can be observed in the case of 48 h (Fig. 5C,F,I,L). It is clear therefore that the PLSR models based on the whole cell average does not accurately or appropriately deal with the subcellular distribution of the radiation effects.

**Figure 6:**
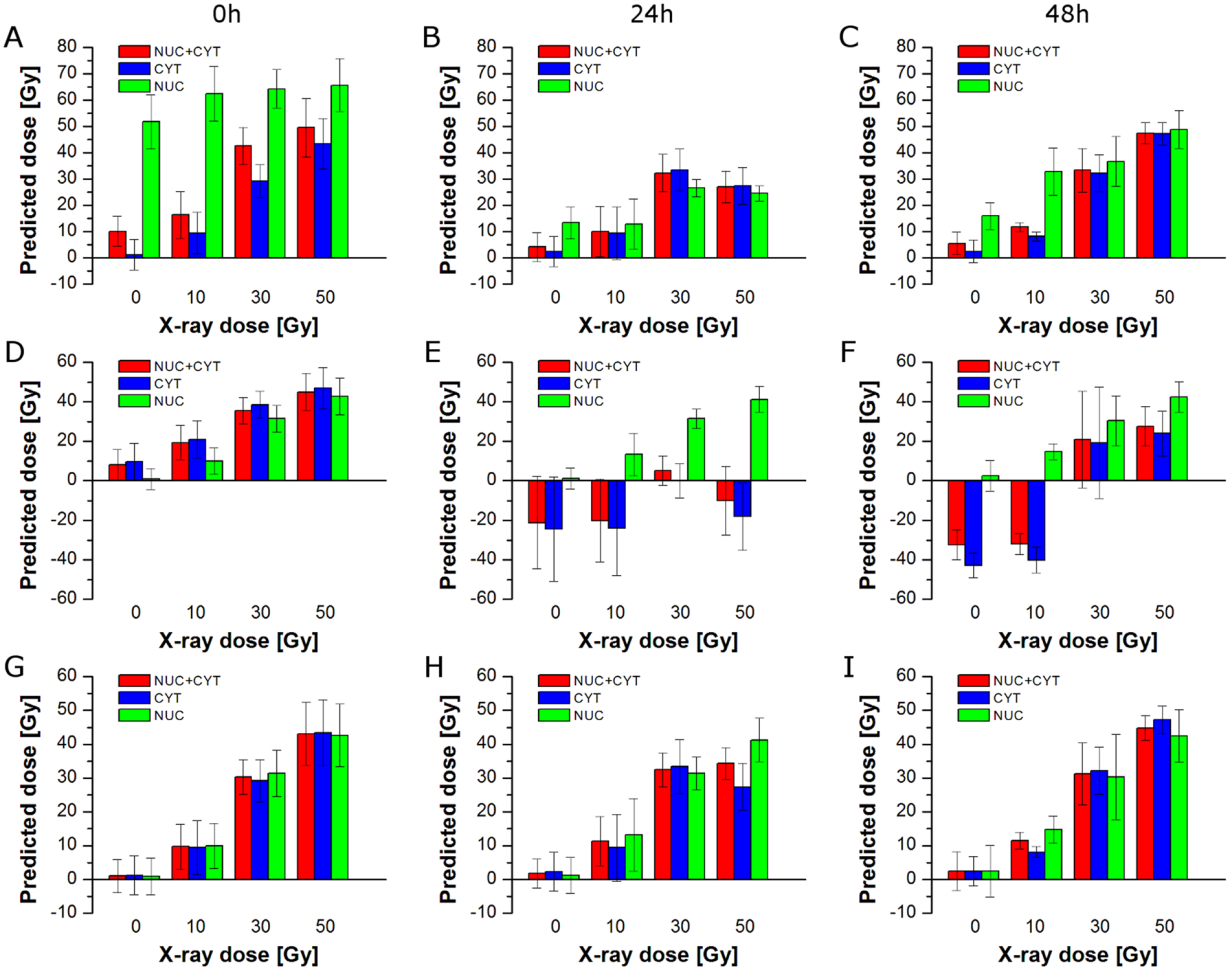
Bar graphs of predicted X-ray dose for 0 h (**A,D,G**), 24 h (**B,E,H**), and 48 h (**C,F,I**) calculated on the basis of the selected PLSR models (PLSR models for cytoplasm (**A**–**C**), nucleus (**D**–**F**), and the whole cell, cytoplasm, and the nucleus (**G**–**I**)) for the average spectra of the whole cell (Nuc + Cyt), cytoplasm (Cyt), and the nuclear region (Nuc).

Figure 5J–L shows differences in prediction of X-ray dose delivered to the whole cell, cytoplasm, and the nuclear region based on PLSR models for the whole cell (see Fig. 1). Figure 6A–C presents predicted doses based on PLSR models for cytoplasm (shown in Fig. 3). As expected, cytoplasm pixels are predicted quite well, while, in contrast, pixels assigned to the nuclear region are not predicted correctly. For 0 h, the nuclear region shows very high (over 50 Gy) predicted dose for all applied doses including control samples (Fig. 6A). For 24 h the prediction is better, although, control samples still exhibit significantly increased X-ray dose (Fig. 6B). For 48 h, too high a dose is predicted for control and 10 Gy samples (Fig. 6C). Furthermore, predicted doses based on PLSR models for cytoplasm show similar trends as observed for prediction based on PLSR models for the whole cell (compare Figs 5J–L and 6A–C). It confirms the dominant influence of cytoplasm pixels on PLSR models for the whole cell. Figure 6D–F presents predicted doses based on PLSR models for the nuclear region (shown in Fig. 4). Good prediction of the responses for nuclear pixels is as expected, whereas prediction of cytoplasmic pixels is problematic. For 0 h, X-ray doses are predicted to be too high for control and 10 Gy samples (Fig. 6D). On the other hand, prediction for 24 h and 48 h turns out to be completely incorrect (Fig. 6E,F). In most cases, predicted doses have negative values, which is unreal, as X-ray dose cannot be negative. It shows that PLSR models for the nuclear region poorly predict cytoplasmic pixels in the studied cells. Figure 6G–I presents predicted doses for cytoplasmic and the nuclear regions based on corresponding PLSR models, *i.e*. doses for cytoplasm were predicted on the basis of PLSR models for cytoplasm (Fig. 3) and doses for the nuclear region were predicted on the basis of PLSR models for the nuclear region (Fig. 4). Finally, doses for the whole cell were calculated for each cell as a mean value of average doses predicted for cytoplasmic and nuclear regions. As can be seen, X-ray doses are predicted correctly in all three cases (Nuc + Cyt, Cyt, and Nuc).

**Figure 7:**
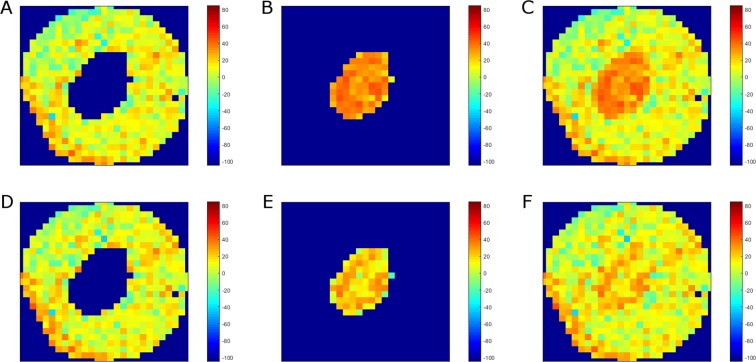
Raman maps of a selected PC-3 cell irradiated with 10 Gy and fixed 48 h post-irradiation calculated on the basis of PLSR models for the whole cell (**A**–**D**) as well as cytoplasmic and nuclear regions separately (**D**–**F**). The maps show cytoplasmic (**A**,**D**) and nuclear (**B**,**E**) regions, and the combination of both regions (**C**,**F**).

Figure 7 presents Raman maps of a selected PC-3 cell irradiated with 10 Gy and fixed 48 h post-irradiation. Figure 7A,B shows Raman maps calculated for the cytoplasmic and nuclear regions on the basis of the PLSR model for the whole cell (Fig. 1E), whereas the map in Fig. 7C is a combination of the two former maps. On the other hand, Raman maps presented in Fig. 7D,E were calculated on the basis of the PLSR models for cytoplasmic (Fig. 3E) and the nuclear (Fig. 4E) regions separately. Furthermore, the combination of these two maps is shown in Fig. 7F. As can be seen, the PLSR model for the whole cell (Fig. 1E) predicts too high X-ray dose for the nuclear region (Fig. 7B,C). On the other hand, as shown in Fig. 6F,I, the PLSR model for the nuclear region predicts the corresponding pixels from the nucleus correctly. It can be also observed in Raman maps shown in Fig. 7E,F. Due to dominant influence of the cytoplasmic pixels on PLSR models for the whole cell, the improvement in prediction of the cytoplasmic pixels is not significant (compare Fig. 7A,D). However, due to clear improvement in the prediction of the nuclear pixels, prediction of the X-ray dose in the whole cell is also improved (compare Fig. 7C,F). The results confirm that it is vitally important to separate pixels from cytoplasmic and nuclear regions in Raman studies on cancer cell response to the ionising radiation, as these compartments are chemically distinct both in composition as well as in their response to radiation.

### Raman mapping *versus* single point measurements

Figures 5–7 show the unequivocal advantage of Raman mapping in subcellular radiation effect studies. Firstly, it should be noted that, any one pixel in the subcellular map contains specific information which is inevitably diluted in either a cellular average or a regression analysis. Two specific examples which should be highlighted are the subcellular distributions of cytochrome C and lipids in the cytoplasm. The analysis of the cellular average indicated that bands at 749, 1587 cm^−1^, commonly assigned to cytochrome C^48,49^, contribute to the irradiation dependent response, although the full spectral response of the molecule is not evident. However, as shown in Fig. S5A (see Supplementary online), Raman spectra from single cytoplasmic pixels differ significantly with respect to bands at *ca.* 750, 1130, 1315, and 1585 cm^−1^. The bands have relatively high intensities at some pixels and can be associated with cytochrome C (compare with Fig. S5B). The presence of cytochrome C is normally associated with the mitochondria and changes in the content and/or distribution of cytochrome C can be correlated with its release after mitochondria damage^49,58,59^. In Fig. 1, the negative contributions of the cytochrome C band in the PLSR β plot for 0 h seems to come from direct damage, whereas positive features for 24 h and 48 h are related with the cell response. However, only bands at 749 and 1587 cm^−1^ are prominent. The absence of other cytochrome C bands in the PLSR β plot can be explained by a much lower intensity of these bands in the average spectra with comparison to the 749 cm^−1^ feature. It can also come from overlapping of cytochrome C bands with bands of other cell components (when bands of other cell components, which concentrations change in the opposite direction and thus have an opposite sign, the intensity of the cytochrome C bands in the β coefficients is reduced).

Other areas of the cytoplasmic region of PC-3 cells exhibited strong lipid features, potentially indicative of the development of lipid droplets (LDs). However, only approx. 10% of the studied cells were found to exhibit such strong lipid bands in Raman spectra of single pixels (see Supplementary Fig. S6). Although the response of LDs to X-ray irradiation is an interesting subject, the number of Raman spectra showing high lipid content was quite limited. The experiments performed in this study were not designed for LDs analysis and a two-micron step size of Raman mapping did not allow a detailed analysis of such subcellular features and a corresponding PLSR model creation. The influence of ionizing radiation on LDs will be followed in a separate study as it requires new experiments to be performed with better spatial resolution to capture a full image of the process.

**Figure 8:**
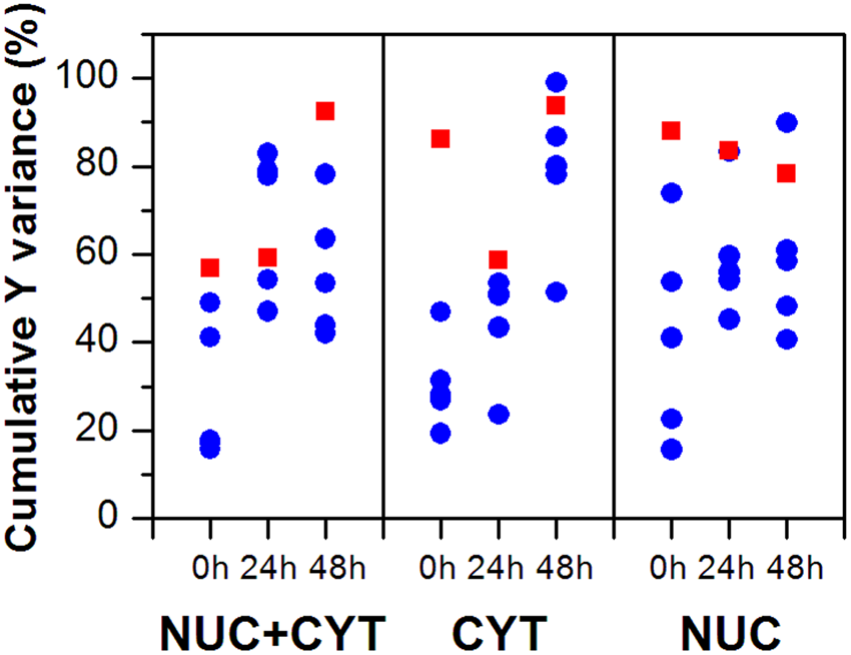
Cumulative Y variance of PLSR models calculated for average spectra (red squares) and randomly selected single pixels (blue circles) of the whole cell (Nuc + Cyt), the nuclear (Nuc), and cytoplasmic (Cyt) regions.

In performing time series or other progression analyses, detailed analysis of regression coefficient plots is necessary to understand the full advantage of the method of subcellular mapping. In the next approach, PLSR models for average spectra (models for the whole cell (Nuc + Cyt; Fig. 1), cytoplasm (Cyt; Fig. 3), and the nuclear region (Nuc; Fig. 4)) were compared with PLSR models for randomly selected (from the existing Raman maps) single pixels (one pixel from the whole cell (Nuc + Cyt), one pixel from cytoplasm (Cyt), or one pixel from the nuclear region (Nuc)). In the first step, the cumulative Y variances of the calculated models were compared to check their predictive power (Fig. 8). As can be seen, in most cases PLSR models based on average spectra (red squares) show higher cumulative Y variance than PLSR models based on single pixels (blue circles). However, some PLSR models calculated for single pixels show higher predictive power than the corresponding PLSR models for average spectra. For instance, the PLSR model for average spectra of the whole cell (Nuc + Cyt) calculated for 24 h post-irradiation reaches 59% of cumulative Y variance, whereas an analogous PLSR model for single pixels with the highest predictive power reaches 83%. However, in the case of the remaining examples, the difference in predictive power is not so prominent (94% *versus* 99% and 78% *versus* 90% for the 48 h cytoplasm model and 48 h nucleus model, respectively). Considering just the Y variance explained by the model may lead to an incorrect conclusion that such models are better. However, it is the regression coefficient and its interpretation that is the most important outcome of PLSR in such studies.

**Figure 9:**
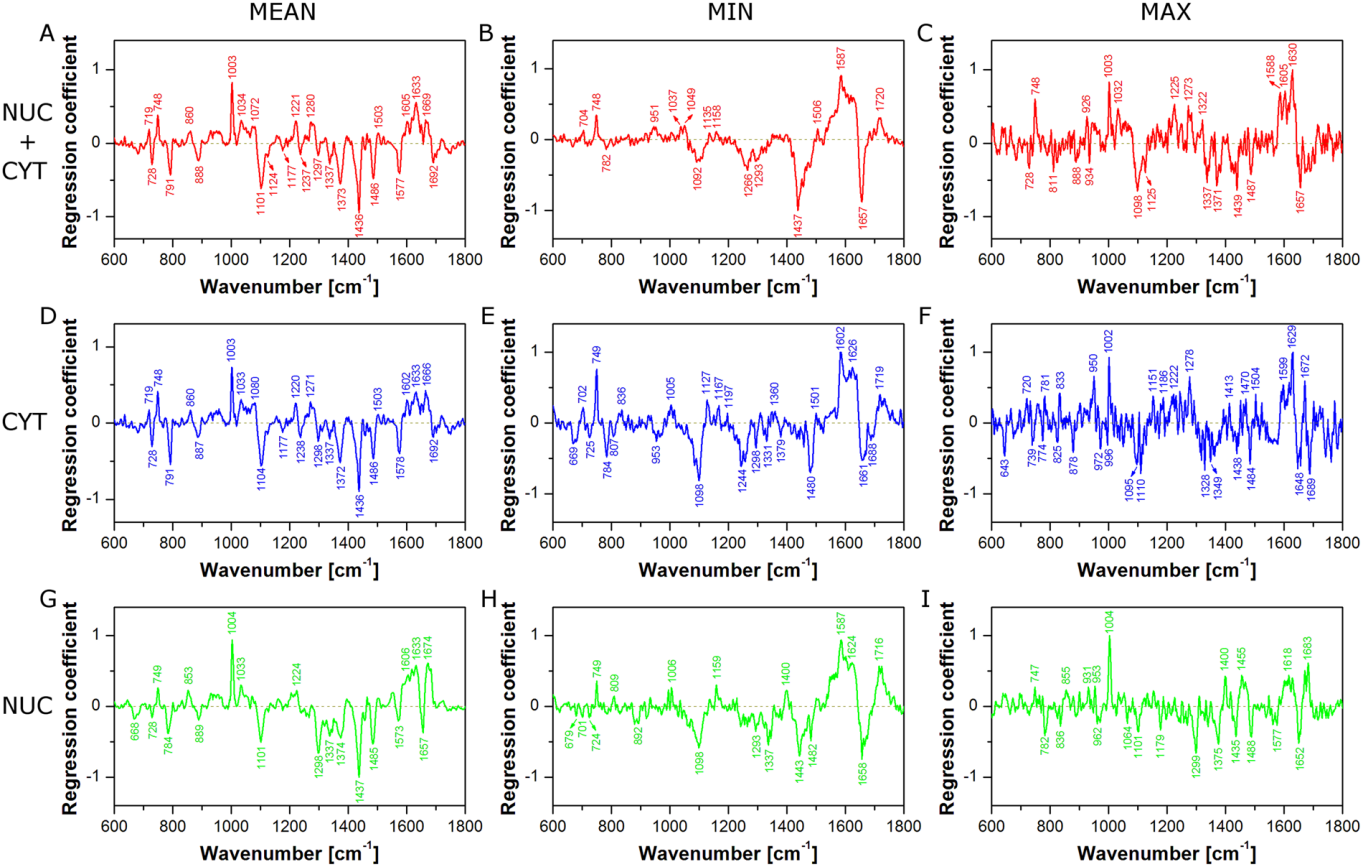
PLSR coefficient plots from models for the whole cell (Nuc + Cyt; **A**–**C**), cytoplasm (Cyt; **D**–**F**), and the nuclear region (Nuc; **G**–**I**) calculated for cells fixed 48 h after irradiation. PLSR models were based on average spectra (Mean; **A,D,G**), randomly selected single pixels with the lowest predictive power (Min; **B,E,H**), and randomly selected single pixels with the highest predictive power (Max; **C,F,I**).

Therefore, in the next step, regression coefficient plots for average spectra (Mean), single pixels with the lowest predictive power (Min), and single pixels with the highest predictive power (Max) were analysed to point out differences in spectral features calculated by corresponding PLSR models (Fig. 9). Figure 9A shows the PLSR β plot for average spectra (Mean) of the whole cell at 48 h. As was described previously (see section ‘Cell response to X-ray irradiation’), some characteristic spectral features can be observed in this plot and assigned to cytochrome C, nucleic acids, proteins, and lipids. On the other hand, Fig. 9B presents the PLSR β plot of the model for single pixels with the lowest predictive power (Min). It is obvious that this plot shows much fewer spectral features than the previous one (compare Fig. 9A,B). Thus, interpretation of radiation effect based on such a model can give an incomplete picture of chemical changes appearing in cells after irradiation. Finally, Fig. 9C shows the PLSR β plot of the model for single pixels with the highest predictive power (Max). This plot exhibits many more spectral features than observed in Fig. 9B. However, some bands are missing (*e.g*. 719 and 1577 cm^−1^). Furthermore, the plot is quite noisy. It suggests that higher predictive power is gained from spectral noise rather than from chemical composition. Analogous consideration can be taken for the PLSR β plots for cytoplasm (Fig. 9D–F) and nucleus (Fig. 9G–I) models. As can be seen in Fig. 9, single point measurements (simulated in our study by randomly selected single pixels) can be risky, because the predictive power of the PLSR model and PLSR β plot quality depends on the measurement position. In the extreme cases our models exhibit either low predictive power and regression coefficient plots poor in spectral features or high predictive power but noisy PLSR β plots. In both cases some chemical information about the actual effects is lost. Thus, PLSR models based on mapping datasets seem to be more reliable and comprehensive.

Raman spectroscopy with the support of PLSR is a powerful tool for analysis of dose-dependent chemical changes induced in cancer cells by ionising radiation. Surprisingly, Raman data obtained for cells fixed immediately after irradiation (0 h) showed clear correlation between applied and predicted X-ray dose. At this point, chemical changes identified by Raman spectroscopy come probably from direct damage caused by irradiation and early cell response. It seems that this spectroscopic method is more sensitive than cell metabolic activity test, which showed no significant effect in cells at 0 h. As expected, Raman data for 24 h and 48 h exhibited good correlation between applied and predicted X-ray dose. Subcellular response of cancer cells to X-ray irradiation was compared for cytoplasm and the nuclear region. Apart from results obtained for 24 h, chemical changes induced by increasing dose of radiation seem to be similar for both cell components. Thus, it appears that cytoplasm and nucleus react in a similar way. However, response strength depends strongly on time and X-ray dose. On the other hand, the application of mapping technique allowed component-specific prediction of delivered X-ray dose, which, to the authors’ best knowledge, is the first report ever. Calculated radiation dose deposited in cytoplasm and nucleus was different within the cells and was dependent on time of cell response (fixation time after irradiation) and X-ray dose. Additionally, prediction of X-ray doses using PLSR models for individual cell components showed that cytoplasm and the nuclear region should be analysed independently. Clear advantage of Raman mapping over single point measurements was confirmed by comparison of PLSR models for average spectra of the whole cell and randomly selected single pixels. In most cases the cumulative Y variance of the models based on average spectra was higher than corresponding variance of the models based on single pixels. However, some models calculated on the basis of single pixels showed better predictive power. Thus, calculation of the cumulative Y variance seems to be not enough to reveal real advantage of mapping technique. Detailed inspection of regression coefficient plots calculated from individual PLSR models showed unequivocally that the PLSR β plots for single pixels can be poor in spectral features and exhibit increased noise level than corresponding plots for mapping datasets. Our results show a strong need for fast imaging techniques in the field of Raman studies (CARS, SRS) on radiation effects in cancer cells.

## Materials and Methods

### Cell culture

Human prostate adenocarcinoma PC-3 cells (ATCC) derived from bone metastases were cultured in RPMI 1640 medium, supplemented with 10% of FBS (foetal bovine serum), 100 U/ml penicillin–streptomycin-neomycin solution, 10 mM HEPES, and 1 mM sodium pyruvate (Sigma Aldrich, Stenheim, Germany). The cell culture was incubated at 37 °C in an atmosphere of 5% CO_2_.

### Irradiation procedure

Cells were seeded on calcium fluoride windows (Crystran Ltd., UK) inside Petri dishes and kept in an incubator at 5% CO_2_ and 37 °C for 3 days to promote adhesion and growth. The cell confluence after that time was ca. 70%. Cells were then irradiated with a single fraction of X-rays at a dose rate of ∼2.1 Gy min^−1^ using a MG325 (250 kV, 10 mA) X-Ray Tube (YXLON, Hamburg, Germany). Cells were irradiated to 10, 30, and 50 Gy. Control cells were left unirradiated. The first set of samples was fixed just after irradiation (0 h) using 3.7% paraformaldehyde in phosphate buffered saline (PBS) for 10 minutes. Other samples were kept in an incubator for 24 and 48 hours and then fixed in the same way. All samples were washed after fixation using PBS (3 times for 2 minutes), PBS/water solutions (2:1, 1:1, 1:2; 2 times for 2 minutes), and pure water (3 times for 2 minutes). Finally, samples were left in the open air over night to dry. All solutions were prepared using ultrapure water (Direct-Q 3 UV, Millipore, USA). The whole experiment (cell culture, irradiation, Raman measurements) was repeated three times.

### Raman microspectroscopic mapping

Raman microspectroscopic maps were recorded using a Renishaw InVia Raman spectrometer equipped with an optical confocal microscope, an air-cooled solid state laser emitting at 532 nm, and a CCD detector thermoelectrically cooled to −70 °C. A dry Leica N PLAN EPI (50x, NA 0.75) objective was used. The power of the laser at the sample position was approximately 7 mW. CaF_2_ windows were placed just under the objective and selected cells were scanned (point by point) using a high speed encoded stage. 5 cells of each sample group (X-ray dose and post-irradiation time) were investigated in each replicate (15 cells for a given set of incubation time and radiation dose). 180 cells in total were studied in this research (15 cells × 12 sample groups). As previously reported^30,31,34^, approximately 20 cells per sample was found to be enough in single spectrum Raman studies on radiation effect on cancer cells. In our study, due to time limits, we reduced the number of cells from each sample group to 15 cells. However, we were able to obtain hundreds of spectra from a single cell. The size of each map depended on the cell dimension, *i.e*. the whole cell area was mapped with a step size of 2 μm. A sum of 3 scans with integration time of 20 seconds and a resolution of *ca*. 1.5 cm^−1^ was collected from each point. The spectrometer was calibrated using the Raman scattering line generated by an internal silicon plate.

### Cytotoxicity

Cell viability after irradiation was assessed via the 3-(4,5-dimethylthiazol-2-yl)-2,5 diphenyl tetrazolium bromide (MTT) assay. For the MTT assay, the PC3 cells were seeded at a density of 10,000 cells/well in 96-well plates 72 h before irradiation. After X-rays exposure, MTT reagent (10 µl, final concentration 0.5 mg/ml, Roche, Mannheim, Germany) was added to each well and the cells incubated for 4 h at 37 °C. The formazan crystals were dissolved in 100 μl of the solubilisation solution (10% SDS in 0.01 M HCl) overnight. DMSO was used as a positive control. The absorbance at 580 nm was measured using a Tecan Spark microplate reader (Tecan Trading AG, Switzerland). The assay was performed in triplicate.

### Data analysis

Before analysis, Raman spectra were baseline corrected (7^th^ order of polynomial), smoothed, and normalised (‘area under the curve’ normalisation) using WiRE 4.2 software (Renishaw, UK). When necessary, a cosmic ray removal procedure was applied. All pixels with phenylalanine (Phe) band (*ca*. 1003 cm^−1^) intensity of less than 30% of the maximal value for the whole cell were discarded as low intensity spectra (pixels on the cell edges, low quality spectra). Thus, three groups of pixels were selected for each cell and used as a dataset (nucleus, lipid droplets, and cytoplasm (without LDs pixels)). Partial Least Squares Regression (PLSR)^60^ and map creation were carried out using MatLab R2017b (MathWorks, Inc., USA). PLS analysis was performed on the Raman spectra with a k-fold Cross-Validation (CV) scheme to determine the optimal number of Latent Variables (LVs) of the model. This was done by finding the minimal CV Error. All chemometric calculations were based on the whole dataset (Raman spectra from all maps). Results shown in Figs 1–7 are based on the PLSR models for average spectra of the whole cell, cytoplasm, and the nuclear region. So each cell was to obtain 3 average spectra and these spectra were independently used in model creation. Figures 8 and 9 contain results obtained using the PLSR models for average spectra and randomly selected single pixels.

## Supplementary information

Supplementary Information

## Data Availability

The datasets generated during and/or analysed during the current study are available from the corresponding author on reasonable request.
