# Supplementary material for: Exploring subcellular responses of prostate cancer cells to X-ray exposure by Raman mapping
Source: Sci Rep. 2019 Jun 18;9:8715. doi: 10.1038/s41598-019-45179-y (PMC6581960; doi:10.1038/s41598-019-45179-y)
Supplement: Supplementary file 1 — Supplementary Information [file 41598_2019_45179_MOESM1_ESM.docx]

**SUPPLEMENTARY INFORMATION**

**Exploring subcellular responses of prostate cancer cells to X-ray exposure by Raman mapping**

Maciej Roman^1,*^, Tomasz P. Wrobel^1^, Agnieszka Panek^1^, Esen Efeoglu^2^, Joanna Wiltowska-Zuber^1^, Czeslawa Paluszkiewicz^1^, Hugh J. Byrne^2^, and Wojciech M. Kwiatek^1^

^1^ Institute of Nuclear Physics Polish Academy of Sciences, PL-31342 Krakow, Poland

^2^ FOCAS Research Institute, Technological University Dublin, Kevin Street, Dublin 8, Ireland

* corresponding author: [Maciej.Roman@ifj.edu.pl](mailto:Maciej.Roman@ifj.edu.pl)

**Analysis of average Raman spectra**

Average Raman spectra of control PC-3 cells and cells treated with an increasing X-ray dose (10, 30, 50 Gy) are presented in Figs. S1, S2, and S3 (for 0h, 24h, and 48h post-irradiation timepoints, respectively).


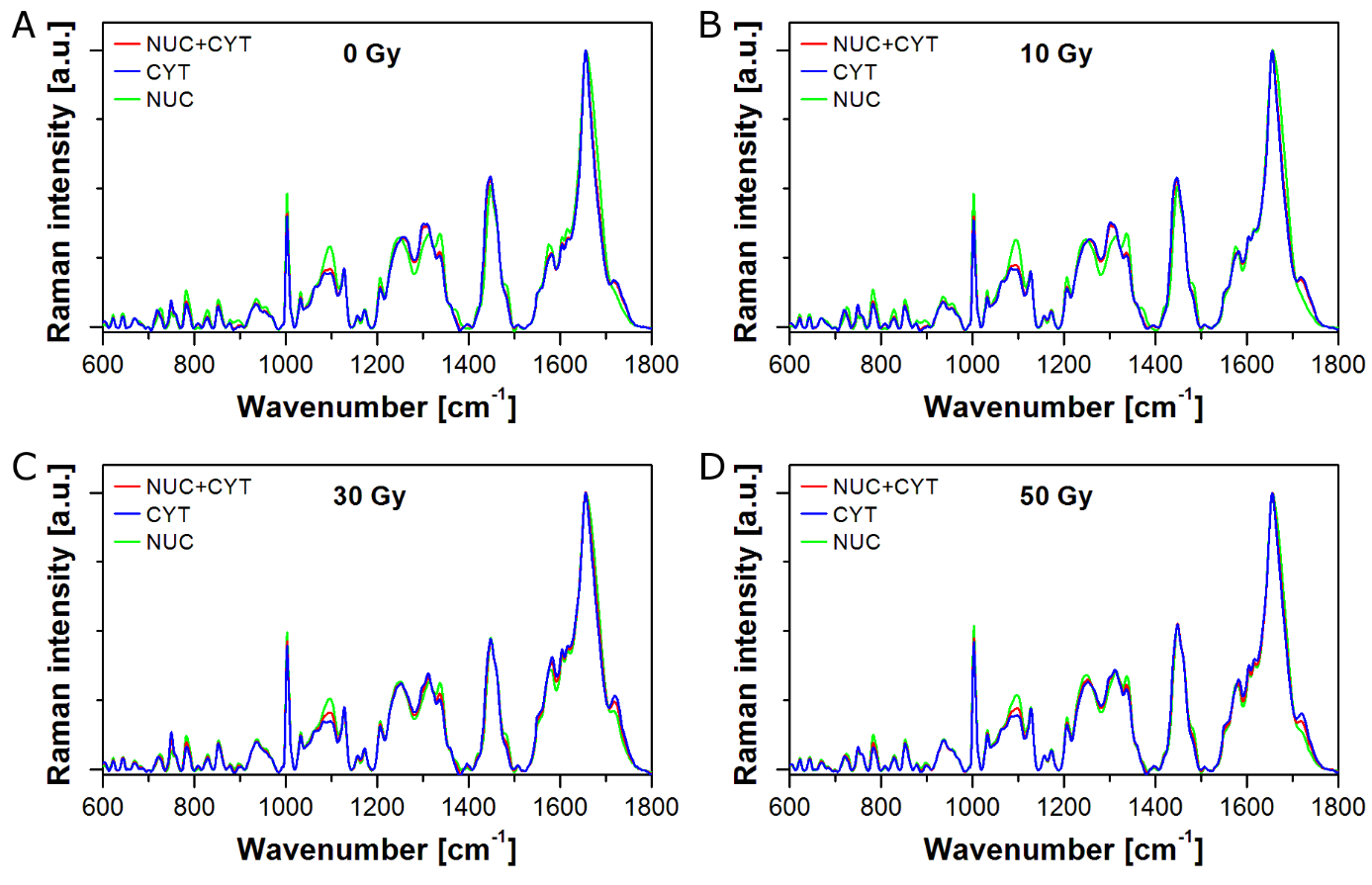


Fig. S1. Average Raman spectra of control PC-3 cells (A) and cells irradiated to 10 Gy (B), 30 Gy (C), and 50 Gy (D) at the 0h post-irradiation timepoint. The spectra are shown for the whole cell (Nuc+Cyt; red line), cytoplasm (Cyt; blue line), and the nuclear region (Nuc; green line).


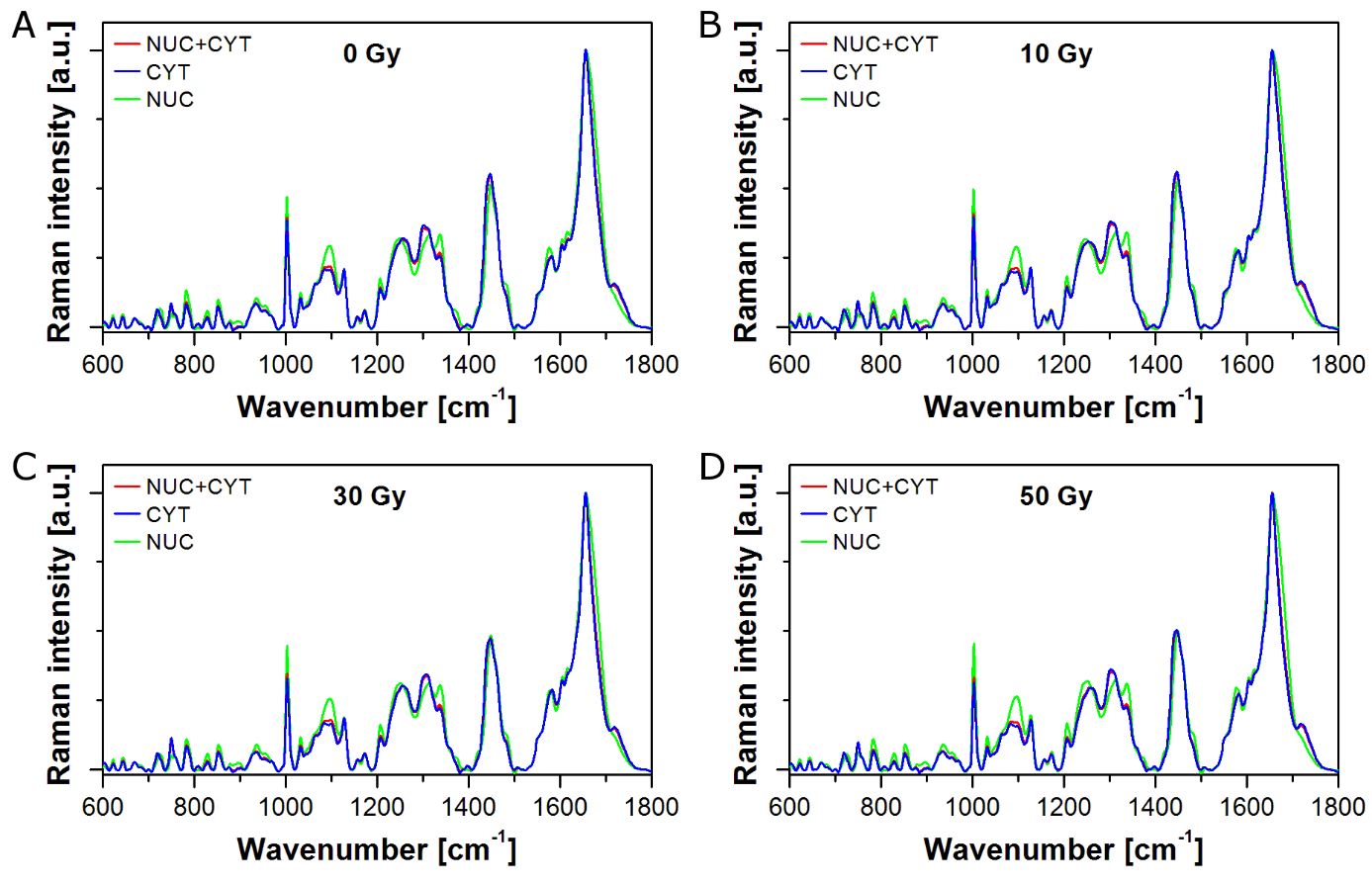


Fig. S2. Average Raman spectra of control PC-3 cells (A) and cells irradiated to 10 Gy (B), 30 Gy (C), and 50 Gy (D) at the 24h post-irradiation timepoint. The spectra are shown for the whole cell (Nuc+Cyt; red line), cytoplasm (Cyt; blue line), and the nuclear region (Nuc; green line).


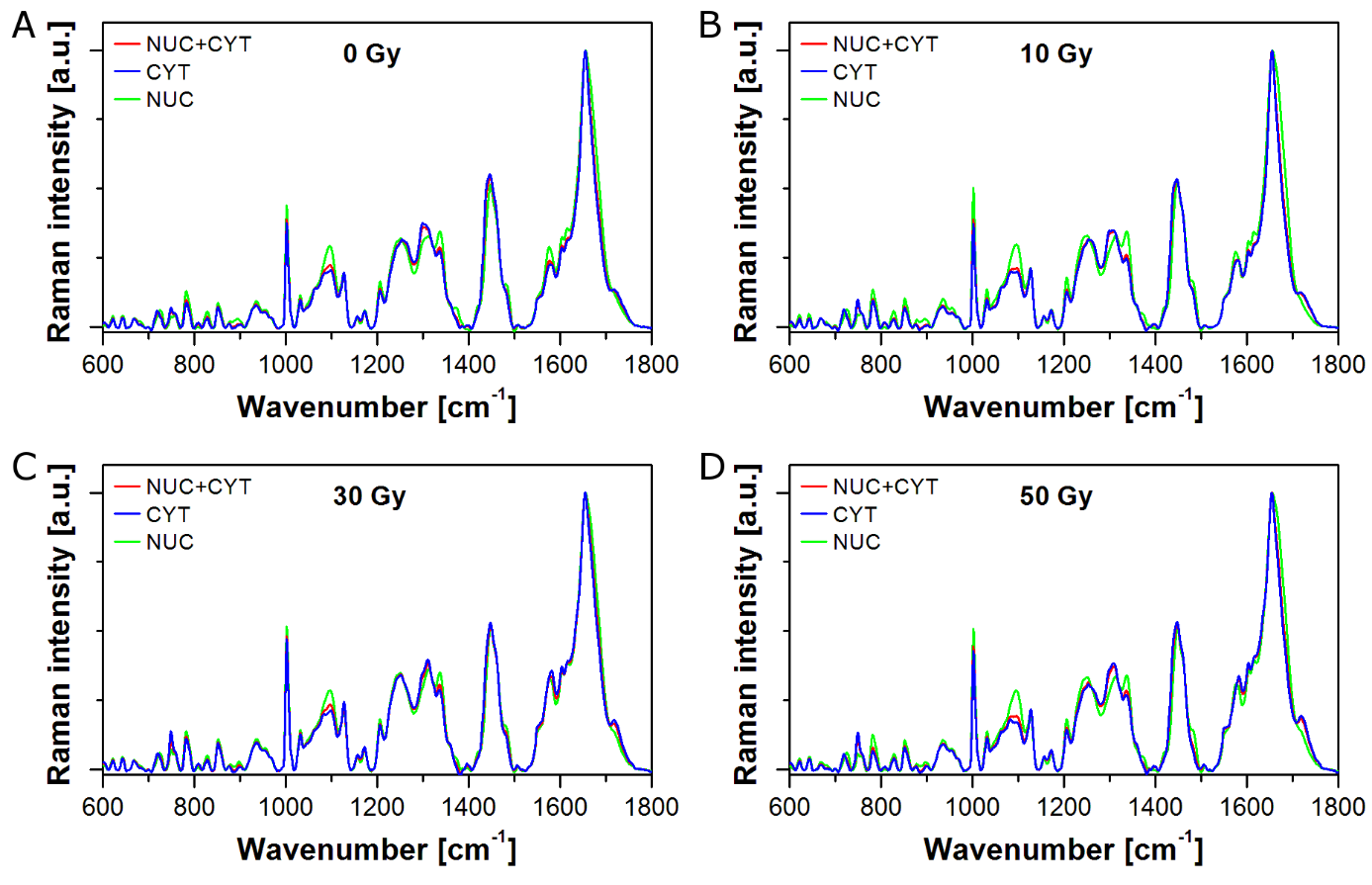


Fig. S3. Average Raman spectra of control PC-3 cells (A) and cells irradiated to 10 Gy (B), 30 Gy (C), and 50 Gy (D) at the 48h post-irradiation timepoint. The spectra are shown for the whole cell (Nuc+Cyt; red line), cytoplasm (Cyt; blue line), and the nuclear region (Nuc; green line).

As can be seen, spectral patterns of the average spectra for the whole cell (Nuc+Cyt) and cytoplasm (Cyt) are very similar for all sets of incubation time and radiation dose. It is a result of a higher number of cytoplasm pixels in Raman maps in comparison with those of the nuclear region (see Fig. S5). As expected, average Raman spectra of the nuclear region exhibit slightly different spectral pattern. The most prominent differences are related to the content of cytochrome C (ca. 750 cm^-1^), nucleic acids (ca. 785, 1095, 1580 cm^-1^), lipids (1200 – 1400 cm^-1^ region), and proteins (ca. 1000 cm^-1^, broadening of the Amide I band). Similar features have been found in the cellular discrimination studies (e.g. Z. Farhane, F. Bonnier and H. J. Byrne, *Anal. Bioanal. Chem.*, 2017, **409**, 1333–1346.). However, the differences between average spectra of PC-3 cells exposed to X-rays at different post-irradiation timepoints are not so prominent and more advanced methods, e.g. chemometrics, must be applied to analyse the induced effects.

**Cytoplasm to nucleus ratios**

Fig. S4 shows examples of optical images (obtained from the spectrometer camera just before the acquisition) and corresponding cytoplasm and nucleus masks calculated on the basis of the characteristic bands of the considered cell regions.


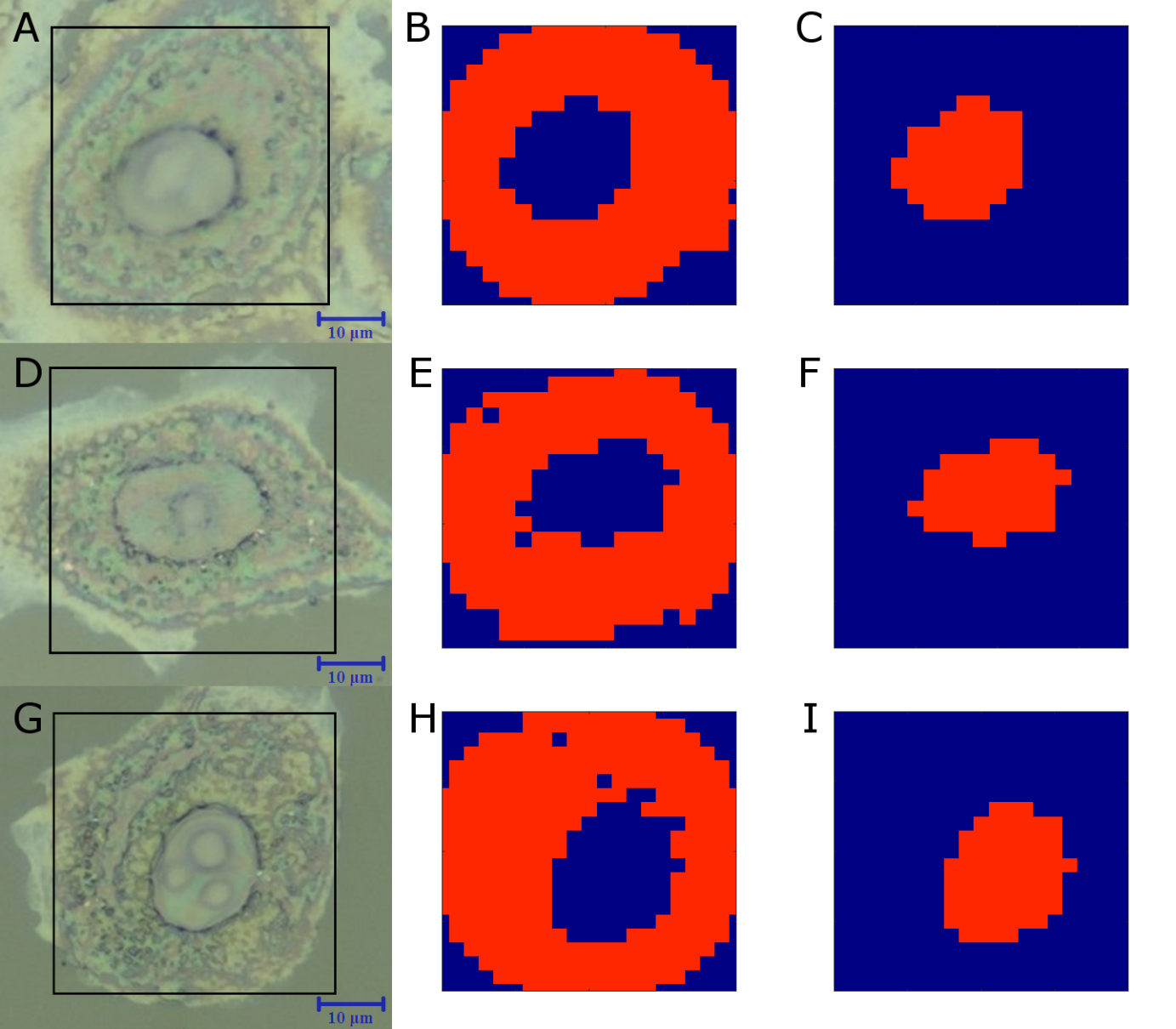


Fig. S4. Examples of optical images (A,D,G) and corresponding cytoplasm (B,E,H) and nucleus (C,F,I) masks of PC-3 cells mapped in this study.

As can be seen, cytoplasm area is much bigger in the studied cells than the nuclear region. Since Raman mapping was performed for the whole cell area with a fixed step size (2 μm), the number of cytoplasm pixels exceeds those of the nuclear region.

**Cytochrome C content**

Raman spectra extracted from single pixels collected from different areas of the PC-3 cell cytoplasmic region are shown in Fig. S5A.


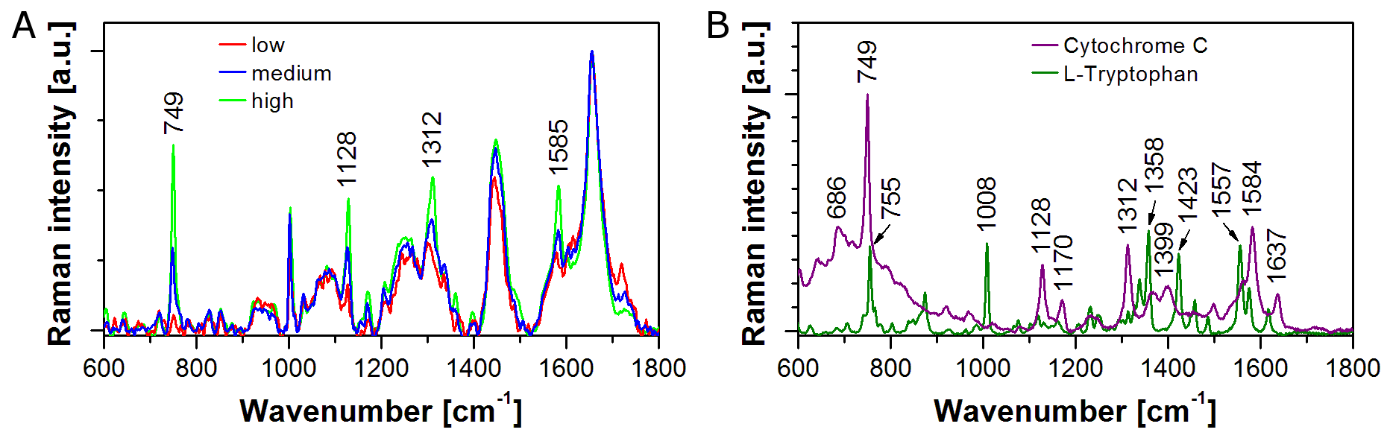


Fig. S5. Raman spectra extracted from single pixels collected from cytoplasmic areas with low (red line), medium (blue line), and high (green line) content of cytochrome C (A). Raman spectra of cytochrome C and L-Tryptophan acquired using 532 nm excitation laser (B).

The most distinct differences between the spectra can be observed at 749, 1128, 1312, and 1585 cm^-1^. All above-mentioned bands originate from cytochrome C, as shown in Fig. S5B, and can be easily found in Raman spectra measured using 532 nm excitation laser due to strong resonance with a heme moiety (see Ref. [48,49]). Cytochrome C is associated with mitochondria presence. Strong cytochrome C bands can be observed only in the spectra from the mitochondrial areas in cytoplasm. On the other hand, L-Tryptophan shows intense bands at 755, 1008, 1358, 1423, and 1557 cm^-1^. Thus, the spectral changes observed in Fig. S5A cannot be associated with an increasing content of this amino acid.

**Lipidic content**

Raman spectra extracted from single pixels containing strong lipidic contributions in the PC-3 cell cytoplasmic region, potentially indicative of lipid droplets, are shown in Fig. S6.





Fig. S6. Raman spectra extracted from single pixels collected from cytoplasmic areas with low (red line), medium (blue line), and high (green line) content of lipids.

The spectra differ in intensities of the lipid bands, *i.e.* bands positioned at 1061, 1099, 1128, 1295, 1440, 1463, 1728, and 1742 cm^-1^. Most such pixels exhibit clear lipid bands of medium intensity together with bands of other biomolecules present in the cell. Furthermore, only some pixels show lipid bands that dominates the whole spectrum. It is a result of Raman mapping with a two-micron step size scanning. Detailed lipid droplets analysis requires measurements with much higher spatial sampling.
